# Palliative care for people with schizophrenia: a qualitative study of an under-serviced group in need

**DOI:** 10.1186/s12904-018-0309-1

**Published:** 2018-03-27

**Authors:** Beverley McNamara, Anne Same, Lorna Rosenwax, Brian Kelly

**Affiliations:** 1 School of Occupational Therapy, Social Work and Speech Pathology, Curtin University, Kent Street, Bentley, WA Australia; 2 School of Medicine and Public Health, University of Newcastle, University Drive, Callaghan, NSW Australia

**Keywords:** Palliative care, Mental health, Schizophrenia, Stigma, Late diagnosis, Communication, Capacity building

## Abstract

**Background:**

People with schizophrenia are at risk of receiving poorer end of life care than other patients. They are often undertreated, avoid treatment and are about half as likely to access palliative care. There are limited options for end of life care for this under-serviced group in need. This study aims to address the paucity of research by documenting possible need, experiences of health care service use and factors affecting palliative care use for people with schizophrenia who have advanced life limiting illness.

**Methods:**

Semi-structured interviews were undertaken with 16 experienced health professionals caring for people with schizophrenia in Western Australia. The interviews focussed on their perceptions and experiences of end of life care, their patients’ unmet needs, palliative care options, and suggested services to support this vulnerable group and improve health care provision. The research used a qualitative design and thematic analyses.

**Results:**

The participants all advocated strongly for their patients and recognised their extreme vulnerability. They identified a range of challenges and unmet needs experienced by people with schizophrenia at the end of life including: illness factors such as the impact of schizophrenia on information processing and communication; social factors such as stigma, isolation and the absence of a carer; and health care factors such as late diagnosis, delayed access to care, and mismanagement in care. Four themes were organised into two domains with the first exploring the individual and social circumstances of people with schizophrenia, including the challenges they experience in the health care system. The second domain covers themes that discuss barriers and facilitators to people with schizophrenia receiving palliative care and key features in palliative care provision, including recognising declining health, communication and planning, and collaboration and capacity building in the broader health, mental health and palliative care sectors.

**Conclusions:**

To ensure people with schizophrenia are well supported at the end of life a model of palliative care is required that offers both specialised services and capacity building within the pre-existing health workforce. Resources are needed to address the stigma and lack of services faced by this vulnerable group.

## Background

Despite its low prevalence (approximately 1%) the health, social, and economic burden of schizophrenia is substantial [1–3]. A schizophrenia mortality meta-analysis indicated that patients with schizophrenia do not receive health care services that are available to the general population [4]. Schizophrenia is a complex disease with level of disability, duration of illness and social costs all contributing to economic costs. Nevertheless, a recent systematic review showed that the substantial economic impact of schizophrenia is suggestive of inadequate provision of health care services to this population [5]. Both the direct and indirect medical and non-medical costs of care for people with schizophrenia are high and strategies are needed to ensure better outcomes for people with schizophrenia, as well as the people who care for them [6].

When compared to the general population, there is a substantially higher incidence and prevalence of morbidity and mortality among people with schizophrenia [7, 8]. A ten to 20 year shortened survival has been reported for this group. Possible multifactorial explanations for the gap include reduced access to medical care, medication side effects, increased prevalence of comorbid illness (eg. cancer, cardiovascular disease and emphysema), increased rates of suicide and individual health behaviours such as increased rate of substance abuse (especially tobacco), poor diet and lack of exercise [3, 9–11]. People with schizophrenia are socially marginalised [8], may experience socio-economic deprivation, stigma, homelessness, and violence [3, 12, 13] and face premature death from preventable conditions [14]. It is particularly concerning that patients with schizophrenia also risk receiving worse end-of-life care than other patients [3, 8, 13].

People with schizophrenia are often undertreated or avoid treatment, particularly when terminally ill [15, 16]. A Canadian study showed that when compared to their matched cohorts people who died with schizophrenia were much more likely to die in nursing homes, were less likely to see specialists (other than psychiatrists) and about half as likely to access palliative care [8, 13]. Another study, which reviewed electronic records and excluded deaths due to accidents, suicide, homicide or undetermined sources, indicated that in the last 30 days of life, people with severe psychiatric illness had reduced inpatient and intensive care unit utilisation, but increased emergency department presentations [17]. Many patients with schizophrenia struggle to report pain or related symptomatology, even with advanced illness [18–20] and receive less opioid analgesia than other patients [3]. Despite the finding that many patients with schizophrenia retain capacity for medical decision making, especially when supported by trusted caregivers, they are less likely to engage in advance care planning [21, 22]. Nevertheless, the symptoms of schizophrenia can have an effect on physical health and the ability to respond to treatments and the symptoms may interfere with capacity to make medical decisions [16].

Given the complexity of their disease and comorbidities, and the stigma they experience, people with schizophrenia with life limiting illness at the end of life have limited options for care. Psychiatric group homes and nursing homes may be poorly equipped, yet palliative care settings may be equally challenged for different reasons. As a result people with schizophrenia are at risk of less than optimal symptom control and unmet psychosocial needs [3]. Although all people are vulnerable at the end of life, people with severe mental illness may face additional barriers from health professionals who may not be adequately prepared to challenge their own assumptions [23]. The vulnerability of patients with schizophrenia must be recognised in palliative care settings so that the care is inclusive and universally accessible [24].

Although there are clear indications that patients with schizophrenia may require palliative care at the end of life, there is a paucity of research that documents possible need, experiences of health care service use and suggestions for approaching care for this vulnerable group. There are two relevant population-based studies [8, 13, 17], but qualitative research is scarce and most often based upon case studies [3, 16, 25, 26]. This study explored the perspectives of health professionals to identify, and help explain, the unmet needs of people with schizophrenia at the end of life and consider what kinds of support they may require. Secondly, it sought to identify the factors which may influence access, as well as barriers, to appropriate palliative care.

## Methods

### Research design, setting and participants

This qualitative study explored the perceptions and experiences of 16 experienced health professionals who were caring for, or had experience of caring for, people with schizophrenia. It focussed on the end of life needs of people with schizophrenia, their experiences with health services and their possible need for palliative care. The health professionals were familiar with people who have schizophrenia, have substantial physical comorbidities and who are approaching the end of life. It aimed to identify both barriers to, and facilitators of, palliative care for this group of vulnerable people in order to provide insights that may strengthen end of life care. The study was conducted in a major city and rural areas in Australia from June 2015 to March 2016. It reports on qualitative semi-structured interviews which were analysed thematically. Health professionals employed in government policy and management, palliative care, mental health, community service provision, clinical care, allied health care, high care residential homes and community-based supported accommodation, including psychiatric hostels, were interviewed.

### Recruitment and data collection

A purposive sampling strategy was used to ensure a range of health professionals with different professional backgrounds working in a variety of public and non-government organisations were included. Participants needed to have either direct experience of caring for people with schizophrenia at the end of life, or supervised other health professionals with direct care responsibilities. The authors used key informants in major hospitals, community organisations and supported accommodation agencies to help identify suitable participants. Participants were initially contacted by either email or phone and then provided with an information sheet which outlined the study aims, requirements of the participants and it ensured confidentiality. The interviews were conducted face-to-face (*n* = 15) or by phone (n = 1) by one of the authors in a place of the participant’s choosing (most often a workplace) and lasted approximately one hour. Three participants who worked in the same location were interviewed together. Each participated fully and the interview lasted two hours. Demographic and personal data were collected from the health professionals to record their position or role; type of organisation where they worked; years of experience; general health professional qualifications; education or training relevant to this population; and their self-rated level of experience of caring for people with schizophrenia at the end of life.

The interview guide was based on a review of the literature. Health professionals were interviewed to explore if there was a need for, ability to access and benefit from, hospital and community-based palliative care for people with schizophrenia and to determine what might influence access as well as barriers to this service. Interviews focused on clinical reality; unmet and met need; and appropriate and inappropriate service provision.

The following are examples of the semi-structured questions used for the in-depth interviews:

1. Discuss your experiences of caring for people with schizophrenia? What things were important as they approached the end of life?
2. What met and unmet needs did they experience?
3. Did they receive palliative care? How did it come about and what changes occurred after receiving palliative care?
4. Did they receive specialised palliative care (from a specific palliative care/hospice service, as opposed to general palliative care often delivered in aged residential care or by General Practitioners) and if so, did they benefit, or not, from this care?
5. Is there a need for specialised palliative care for this group of people, and what form could that take?
6. What is needed to access appropriate palliative care or end of life services?
7. What would prevent access to palliative care or end of life services?
8. What policies are in place at your work for palliative care or end of life services?
9. Provide comments on what would improve end of life care.

### Data analysis

Simple descriptive analyses were used for the demographic and personal data. All other data were de-identified and interview audiotapes were transcribed. Transcripts were analysed using a thematic qualitative approach with broad themes identified from readings of the transcripts. Similar narratives were grouped and themes were developed that represented the views, observations and understandings of the health professionals. Two of the authors independently coded the data and preliminary categories were revised. Thematic analyses of the transcripts were discussed and verified by the co-authors. A framework was developed that focussed first on vulnerability and the challenges faced by people with schizophrenia at the end of life; and second on barriers and facilitators to people with schizophrenia receiving palliative care.

### Ethics

Ethical approval was granted by Curtin University Human Research Ethics Committee (APP 1084890), The WA Country Health Service Human Research Ethics Committee, The Department of Health WA Human Research Ethics Committee and Silver Chain Human Research Ethics Committee. Informed consent was obtained prior to the interviews with the participants. All data were stored in a password protected folder with only the investigators having authorised access to the data.

## Results

All participants in the study had some level of experience with either treating, or caring for people with schizophrenia, or had background knowledge about health services for people with schizophrenia through supervision of other health professionals. There were eight nurses, three social workers, three occupational therapists, a counsellor and a psychiatrist in the sample. They worked in a variety of roles which included managers and directors of care, clinical consultants and specialist mental health and palliative care nurses. Three of the participants worked specifically in a role which covered both mental health and palliative care. Work places were both public and private and included a community palliative care organisation, mental health accommodation services, public hospitals, a psychiatric hostel and a country health service. The participants’ years of experience ranged from four to 30 years. The majority had at least 10 years of experience, with seven of the participants having worked in a related area for 20 or more years. Most of the participants had completed specific education or training in either palliative care, mental health, or both. Three of the participants rated themselves as being very experienced in the area of care, eight rated themselves as somewhat experienced, three felt they were not very experienced and one did not provide an answer.

The participants all advocated strongly for their patients and recognised the extreme vulnerability of those they cared for, particularly as their patients life limiting illnesses progressed and they neared the end of life. All of the participants identified a range of unmet needs experienced by people with schizophrenia at the end of life and attempted to explain why they believed this to be so. The study themes have been organised into two domains with the first exploring the individual and social circumstances of patients with schizophrenia, how these patients experience the health care system and the interrelationship between all three factors. The second domain covers the barriers and facilitators to people with schizophrenia receiving palliative care and the benefits of receiving this kind of supportive care. Where the participants own words have been used in quotes their professions are noted in order to provide background about the form of care they provided.

### People with schizophrenia at the end of life: challenges of a vulnerable group

#### Individual factors that may affect people with schizophrenia at the end of life

The participants reported that the experience of people with schizophrenia at the end of life varies dependent upon individual factors such as personal history, a fluctuating mental state which may be influenced by a progressive illness and co-morbidities, compliance with medication and the side-effects of changing medications. Medications, including antipsychotics, are often reduced or changed dependent on the person’s physical disease and some support staff in palliative care or mental health care services reported that they, or their colleagues, find this challenging. Many patients with chronic schizophrenia take Clozapine, a pharmacologically complex drug which, when combined with other treatments and medications, can lead to unwanted and possibly dangerous side-effects. It was reported that staff unfamiliar with patients with schizophrenia experiencing physical decline at the end of life may not recognise the signs indicating the patient is in distress.

The issue of information processing and communication (eg. understanding of the problem, decision-making, communication) was identified as a particular concern. People with schizophrenia as well as a progressive life limiting illness are often challenged as their physical illness progresses. A psychiatrist specialising in palliative care explained:

There are shared challenges around decision making and the ability to weigh up complex information, provide informed consent to different sorts of treatments…they may have challenges with self-care...and little insight about their needs to live safely at home, and so that just gets exacerbated when you have someone who also has a substantial physical illness and who’s transitioning to an end of life phase of that illness, because often people desperately want to stay at home.

#### Social factors that may affect people with schizophrenia at the end of life

It appears most people with schizophrenia at the end of life are vulnerable due to social isolation and marginalisation, which many will have suffered most of their lives. The nature of their mental illness and the manner it plays out in social settings means that people with schizophrenia are often misunderstood and stigmatised. A nurse from a psychiatric hostel explains:

Our residents with schizophrenia have a very, very colourful inner-life which expresses itself in behaviours which I guess would be difficult for the general public or the general community to understand, accept or deal with.

When asked what was the most important factor in receiving good end of life support for people with schizophrenia many participants answered ‘strong social support’. Not having strong external social supports and possible prior family estrangement means that the person with schizophrenia can lack a strong advocate to help navigate the complex end of life trajectory. Unless they have an assertive carer, be that a paid professional or a family member or other friend, they are unlikely to be provided with optimum end of life care. Those most at risk may be homeless or living on their own in a boarding house type of accommodation.

You have to take into consideration that because of their life long conditions, their view of the world, their need for support and a familiar environment could be quite different from somebody who has their cognitive facilities and who has a really strong social support network…they’ve got quite fractured social networks and sometimes the families that are hanging in there may or may not be advantageous to the client at times (mental health nurse).

#### Health care factors that may affect people with schizophrenia at the end of life

Participants reported that one of the greatest challenges for people with schizophrenia receiving good end of life care is a late diagnosis of a life limiting physical disease, like cancer, advanced heart disease or emphysema. The person themselves may avoid going to the doctor based upon their previous experiences which they may perceive as unsatisfactory or even threatening. When they do present, perhaps through a referral ‘late on a Friday afternoon, with members in a shared house unable to support the person’ (palliative care nurse), or through an emergency department in a public hospital, it may either be too late for the person to receive adequate care, or they may be misdiagnosed or mismanaged.

One of the big issues will be the service provider’s understanding of what’s going on for that person. If there is not an effective understanding…it may lead to decisions being made that are inappropriate...a person may be judged on their behaviour at a particular point in time which could lead to a range of consequences around whether a service is provided or not. So if a provider was to front up to the person who was very aggressive that will of course lead to a whole range of requirements in terms of how that’s managed (social worker, support manager in a community-based palliative care service).

End of life care and a death in hospital seems to be the default position for people with schizophrenia in Western Australia who are believed to be ‘in the too hard basket’ (palliative care social worker and government administrator). Most of participants reported that adequate care and referral to a palliative care team is based upon the presence or absence of a strong advocate, most likely a case manager who has a history with the client. Additionally all of the participants spoke about the importance of bringing a multidisciplinary team of experts together to adequately assess, treat and support the patient (and their family, if they are present). However, many of the participants noted that hospital-based and care staff are under-resourced and are often not aware of the needs of people with schizophrenia as their physical health fails.

We’re seeing some dying in hospital because no-one else knows where to put them. A hospice might see schizophrenia and think “hmm a bit hard to manage in the hospice environment”… have to be in a hospital where they potentially might need a guard…if they come from the (name) Mental Health Hospital…they’re prison sentences there, so they’re dying in custody. They’ll all automatically be sent to hospital to die because they’re death in custody so they’re potentially not even thinking about a hospice situation (palliative care clinical nurse consultant).

Even when there appears to be a willingness to support people with mental illness at the end of life, the health care system is not prepared and a siloed approach to care continues, as a palliative care senior social worker explains:

They have fallen between the cracks for the different funding programs so again it’s not been through, I guess, a lack of willingness or preparedness on the part of service providers but quite often it’s then finding the right sources of funding to be able to meet their needs.

#### The interrelationship between factors that affect people with schizophrenia at the end of life

**Fig. 1:**
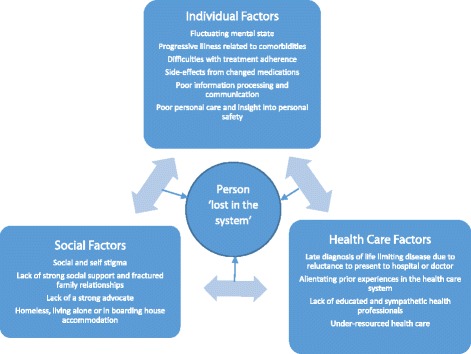
The interrelationship between factors that may affect people with schizophrenia at the end of life

According to many of the study participants, individual, social and health care factors are all interrelated. Figure 1 depicts a set of factors that impact one another in the lives of people with schizophrenia at the end of life. Alienating prior experiences with the health care system, limited cognitive capacity, escalating physical symptoms, social and self-stigma, and assumptions made by health professionals who may lack education, sympathy and resources lead to people with schizophrenia being ‘lost in the mainstream system’ (nurse, general manager in a community-based palliative care service). Without support the person may be overwhelmed by the challenges they face.

### Barriers and facilitators to people with schizophrenia receiving palliative care

It was clear that the participants recognised the range of unmet needs of their patients with schizophrenia at the end of life, with all agreeing that specialist palliative care was necessary for this vulnerable group of people. However, they also acknowledged that being referred to palliative care and receiving palliative services was not straightforward, particularly if people with schizophrenia were ‘lost in the system’, without a strong advocate or without a secure place to live. Table 1 uses data from the participant interviews to provide an overview of the barriers, facilitators and outcomes associated with palliative care for people with schizophrenia at the end of life. As noted by the participants, many of the elements required for palliative care would serve for any person at the end of life as all people are vulnerable as they approach death. However, there are features specific to people with a history of severe mental illness. As noted, information processing and communication problems (some participants used the term ‘limited cognitive capacity’) and the lack of an identifiable carer puts the person with schizophrenia at particular risk. Early referral to palliative care is very important for this group so that ‘there’s trust developed and a good understanding between the client and the services’ (palliative care occupational therapist).

**Table 1:** Barriers, facilitators and outcomes associated with palliative care for people with schizophrenia

Elements required for palliative care	Barriers	Facilitators	Outcomes
Person-centred, individualised care	Problems with information processing and communication Possible cognitive impairment (particularly when paired with mobility) Significant comorbidities Possible lack of insight	Ensure sufficient time to discuss end of life issues Provide adequate symptom assessment and management Provide sympathetic support	Symptoms controlled Anxiety reduced Comfort ensured Level of autonomy encouraged
Identifiable carers	No identifiable carers in the home environment (eg. sharehouses, living alone, homeless) Previous family disruption	Identify or nominate carers (institutional or community agency care staff may be defacto family) Public advocates if needed	Patient supported
Health workforce prepared	Lack of experience Ill-informed assumptions about schizophrenia Lack of adequate educational resources	Ensure adequate training and exposure	Reduction in ignorance and anxiety
Appropriate place of care	Assumptions that the patient will be unmanageable Paperwork seen as a deterrent, especially for short term residence	Identify and update databases of appropriate and willing agencies if transition is required Ensure place is appropriate for the age and individual needs of the patient	Patient supported in a safe and familiar place
Early referral	Delays in patient seeking care because of pain-perceptive and pain-processing abnormalities. Patient or family resistant to presenting to services Health professionals not identifying symptoms and referring appropriately Services not designed to facilitate early referral (ie. response time slow, bureaucratic approaches)	Enlist mental health liaisons and advocates where they are available Educate General Practitioners Coordinate medical and psychiatric systems of care	Adequate symptom control Better end of life preparation Development of trust between patient and team and between services
Continuity of care	Lack of resources to keep patients in a palliative care service when their physical symptoms may appear temporarily controlled	Ensure good medical care Identify advocates Support staff to keep patient in a familiar environment	Holistic care provided Symptoms controlled Familiarity and comfort
Safe supportive place to die	Not dying in a place of choice Lack of identifiable home Inability of institutional or community care staff to provide adequate care Lack of sufficient resources	Support staff to keep patient in a familiar environment Create a home like environment when transitions are required	Familiarity and comfort A good death
Multidisciplinary team	Not all members of the team readily available, with sporadic involvement	Establish regular case conferencing Identify lead or team member responsible	Holistic care provided
Collaboration with other health care and/or community services	Resistance to shared ways of working	Establish case conferencing Include mental health case workers or General Practitioners in the palliative care team Share lead roles between palliative care and mental health workers depending on the patients’ symptoms	Partnership models established Strong involvement of all stakeholders Better quality of care Greater cost efficiency
Family conferencing (when appropriate)	Previous family disruption Lack of prior possible mediation or counselling to prepare	Ensure robust preparation, (eg. questionnaire to identify what should be included or avoided)	Patients and families supported Problems explained and anticipated Families empowered
Risk management	Services and staff unprepared Lack of policies or guidelines Fear and ignorance of staff	Make sure staff are sufficiently trained to recognize when the safety of the patient or other residents may be compromised	Creation of a safe place of care
End of life wishes/ advanced directives	Lack of a family member, identifiable carer or advocate	Identify an advocate (public or otherwise) Ensure continuity of care	A dignified death
Bereavement support	Difficulty in identifying those most affected Previous family disruption Lack of understanding of the grief of others in institutional or community settings	Establish inclusive funerals and memorials Provide support for informal and health professional carers	Families and care staff supported
Capacity building	Lack of communication between teams Lack of resources	Ensure the palliative care team do not ‘take over’ but build capacity within the pre-existing care team	Care staff supported Opportunities for ongoing education

#### Recognising declining health, communication and planning

The participants reported that a key to good palliative care is early recognition of declining health and a gradual acceptance of death. However, people with schizophrenia may not identify the significance of symptoms indicating decline in their own health. Additionally family, informal carers and even staff in psychiatric group homes or nursing homes may not recognise the declining health of the person. A number of factors combine to determine if the person has support to receive palliative care and prepare for death. ‘Some teams are not proactive’ (community mental health nurse), General Practitioners may have limited contact with the patient, family may be resistant to discussions, and the person themselves may be socially isolated and not live in some kind of supported accommodation. As a palliative care nurse consultant stated: ‘I think they are pretty well at the mercy of who they see and when they see them’.

Alternatively, once a palliative care team is in place, the patient will have a much better outcome:

Our residents are reviewed by the GP to confirm any declining health issues or their health status…so whether it’s loss of appetite or weight loss or just through the medical investigation that might warrant a palliative care approach. That is then communicated with a multi-disciplinary team and in particular our palliative care link team which we have formulated and this is to inform everyone, make sure everyone’s on the same page and therefore we would tailor their individual palliative care approach, their needs and preferences (occupational therapist working in mental health and aged care).

Participants spoke about how open communication leads to clearer decision making, careful care plans and appropriate advance care planning. Decisions involve advanced health care directives, enduring powers of guardianship, enduring powers of attorney, wills and funeral arrangements. Place of care and desired place of death are also important topics of discussion. Most of these topics can be approached in well-targeted case-conferencing with all of the providers and the person (and/or their carer) present at the same time. Some of the participants noted that it is also important to acknowledge the needs of carers and the work they do. Key to all of these processes is the presence of a strong advocate, in the form of a family member, friend, health professional like a General Practitioner or mental health professional, or a public advocate. Participants felt that good communication with the appropriate people led to allowing the person with schizophrenia to have choice and to die in a safe, home like environment. It is also important to resist a funding system that requires speedy decision-making and take the necessary time to assess the person properly as there is a risk of under-detecting the problems when the person is unable to express their needs clearly.

The people that know them can advocate on their behalf as long as we try and move away from that labelling principle…you need a really well documented support plan of how to approach the person…what are their usual routines, what sort of indicators, what’s their normal behaviour like so that you can judge what they’re doing or not…it’s very much about identifying what are the goals of care…what are the person’s key goals and so negotiating a lot of that right up front so not waiting for the moment when something really goes awry and then you don’t have a contingency plan (mental health nurse).

#### Collaboration and capacity building in the broader health, mental health and palliative care sectors

All of the participants noted that caring for people at the end of life requires a team approach. They spoke of how the team must unite within an organisation and requires a multidisciplinary approach to ensure that the physical, psychological, social and spiritual needs of the person are taken into account. Equally so the complexity of care required necessitates collaborative approaches between different components of the health care sector. As referrals to palliative care may take place from within general practice, outpatient services, emergency departments, general hospital wards, mental health hospitals, correctional facilities, supported accommodation, homeless shelters and aged care providers, capacity building must take the form of strong advocacy, education and increased resources. A small number of the participants included education of the workforce as a primary concern, including the palliative care workforce, to reduce the stigma associated with severe mental health conditions and to build practical knowledge. In any situation where the person with schizophrenia has pre-existing support it is important to acknowledge this.

I think any supported residential environment throws up a whole range of specific issues because then there’s already a pre-existing workforce with its own organisation so that needs to be understood and there really does need to be a shared understanding about who’s doing what and why…so that does bring up a whole range of practical issues just in terms of gaining access, documentation, all of that sort of thing, so that needs to be an open transparent shared care situation (palliative care social worker).

The introduction of palliative care must be done sensitively and palliative care teams must also be prepared as they may not have the required skills:

There’s a lot of issues I think around how well equipped health professionals generally are to work with people with a range of mental health issues in a community setting and often health teams are not necessarily the best equipped. So sometimes within a palliative care setting essentially you’ve got a group of health professionals whose expertise is working with end of life issues, complex palliative care issues but that may not automatically translate into being able to work effectively with people with mental health issues (palliative care consultant nurse).

Participants noted that collaborative models of care are required that build capacity within existing services but also introduce specialised palliative care to ensure appropriate care is provided. Some participants were aware of approaches used in other parts of the world and felt Western Australia needed upskilling in this area. However, they were also conscious that the numbers of people with schizophrenia at the end of life were not as great due to a smaller population. Nevertheless, all acknowledged that resources were needed in this area.

Doing that multi service really integrated care, and there’s no reason why you couldn’t do this in the same way. So if it was a client with schizophrenia that they actually had their dedicated mental health worker as part of that team, so they work collaboratively with the community palliative care service, and their kind of health worker, maybe their GP and family, so again you’ve got this really nice partnership model. It’s not that hard but it’s different and I think this is where lots of services struggle…It’s easy you just have to think differently (palliative care nurse).

## Discussion

People with severe mental illness at the end of life have as much right as anyone else to have their physical, psycho-social and spiritual needs addressed [27]. Nevertheless, the participants in our study have provided accounts that indicate that many people with schizophrenia at the end of life face numerous challenges in achieving this goal. These include a lack of social supports and strong advocacy; problems with information processing and communication; late diagnosis of a life limiting physical illness; and alienating experiences with the health care system. The health workforce in Western Australia is under-equipped to face the challenges, and while many may be willing and have the best intentions, poor resourcing and lack of education obstructs their path. It appears that interrelated patient-based, provider-based, and systems-based factors are influenced by mental health stigma and may impact the end of life care of people with schizophrenia [28]. People with schizophrenia may internalise perceived prejudices and develop negative feelings which can lead to lack of adherence to treatment [29, 30] and delayed access to appropriate care. When combined with their psychiatric illness, the self-stigma may impede quality outcomes in care. However, it is the stigma encountered at a provider and systems level that is most obstructive in achieving good end of life outcomes for people with schizophrenia. Evidence provided in this study, and in the literature that supports it, indicates that people with schizophrenia at the end of life receive sub-optimal care when compared with the general population. Strong advocacy from within the palliative care and mental health communities is needed to challenge this entrenched stigma.

### Implications for practice

Health professionals who work with people with schizophrenia at the end of life may find the care both challenging and rewarding. As patients with schizophrenia may fail to recognise their own physical decline, determining decision-making capacity is a difficult and ethically fraught step. Stereotypes may arise from presumed incompetence and the fear that end of life discussions may be destabilizing, yet there are tools and methods available to help [23]. A diagnosis of schizophrenia does not mean that person is unable to make a decision about their medical care [29]. However, if a patient is unable to give consent to treatments, or to ceasing treatments, they will need a health care proxy. Using a collaborative approach will assist health professional carers to enlist the support of colleagues with expertise in this area. If the person with schizophrenia does not have an identifiable and reliable support person, such as a trusted family member or friend or a pre-existing advocate, a public advocate must be appointed to assist with decisions. Ideally this should be done as early as possible as appointing someone with no history of the person or relationship with that person is not ideal.

Unfortunately there is evidence to suggest that patients with severe mental illness receive less than adequate care [3, 4, 8, 13]. An American study showed that in many study samples with severe mental illness, rates of guideline adherence were considerably lower than estimated rates for the overall US population [31]. There are guidelines available which promote thorough physical and psycho-social assessments for all people with severe mental health problems. These require practitioners to take into account medication effects, lifestyle issues, pre-existing and developing conditions, alcohol and illicit drug use and psychosocial issues [32]. Assessments of people with schizophrenia at the end of life must be thorough and not rushed. A number of participants in the study noted that problems can go undetected and the person may become lost within a system that is not designed to manage complex patients with psychiatric and physical illness.

Palliative care services for people with schizophrenia must adopt an inclusive approach. Inclusive and accessible palliative care will require creative approaches to include people who are mentally ill in traditional and pre-existing settings through coordination with other care services [24]. A capacity building approach helps health professionals in various settings, including palliative care staff, general practitioners, mental health nurses and accommodation staff, to challenge their own assumptions about people with mental illness at the end of life and build skills in supporting this vulnerable population. General practitioners, in particular, can act as a bridge between palliative care and mental health services [27] and their education and upskilling is important in supporting people with schizophrenia at the end of life.

This study focused on schizophrenia as it is a highly disabling and stigmatized condition affecting a small number of people. We chose to focus on people with schizophrenia as they will most likely have a complex history which may make them reluctant to engage with health care services. Their reluctance together with possible stigma and misconception on the part of health care professionals creates unique challenges for palliative care providers. Many of the findings from the study can be translated to people at the end of life who have other long term severe mental health conditions. A history of alienation, marginalisation, stigma, problems with information processing and communication may also be evident in patients with bi-polar disorder, psychosis or intellectual disability. Psychosis may also be temporary but aggravated when a person with dementia or severe depression has multiple co-morbidities at the end of life. Equally so, homeless people who may or may not have severe mental illness may require particular attention from palliative care providers, including the provision of appropriate services in the place of the person’s choice. Many of the suggestions outlined in Table 1 may be relevant to these groups, but must always be used at the discretion of the treating clinician. As with any patient with complex co-morbidities, medical clinicians must also be aware of the pharmacological implications of introducing new medications to people with schizophrenia.

### Study limitations

The sample size for this study was small due to the overall comparatively small population of Western Australia. Schizophrenia prevalence is relatively low and therefore specialist palliative care workers may not be familiar with caring for patients with schizophrenia. Likewise, mental health workers may not have had a high level of experience with people with schizophrenia at the end of life. We attempted to address this problem by a purposive sampling or a ‘nominated expert sampling’ [33] method that targets people most likely to have had experience in caring for people with schizophrenia at the end of life. Nevertheless, only four of the participants rated themselves as very experienced in this area. Of the three people who rated themselves as not very experienced, one was new to her job and the other two worked in managerial roles and their clinical experience was dated. Qualitative studies with careful sampling designs help resolve validity issues. Three of the participants were interviewed together due to practical reasons. We were aware they may have been influenced by the responses of other participants present, so interviewing techniques were used to ensure each participant was able to provide detailed responses to questions and elaborate where required.

Qualitative studies do not seek to generalize their findings, but aim to provide a level of validity whereby others can transfer ideas from one setting to another [34]. Our study aimed to provide this level of transferability, particularly for other settings where there is universal health care coverage. Even relatively small qualitative studies may achieve validity and transferability through careful sampling, reference to a broad range of literature, a well-constructed research design, a transparent interview protocol and theoretically based analysis [34].

We attempted to include people with schizophrenia approaching the end of life, or their family or informal carers (including bereaved carers) in our study, but this was not possible due to a number of practical issues, including a prohibitive level of gatekeeping amongst the organisations approached. Further studies, with greater numbers of participants, including patients, family members and carers is recommended.

## Conclusion

This study has built on previous literature to confirm that Western Australian people with schizophrenia at the end of life are at risk of not receiving good medical and psycho-social care, including palliative care. They have complex needs that challenge the general health workforce as well as the more sympathetic practitioners in mental health and palliative care. Barriers to palliative care have been outlined, but more importantly facilitators to palliative care have been presented that identify key indicators needed to ensure good care. These include identifying a carer or nominating an advocate for the person, case conferencing in multidisciplinary and cross sector teams, and building capacity within the pre-existing workforce and care providers through education and advocacy. Inclusive and collaborative models of care are required and resources should be allocated to both specialised palliative care and mental health services so that people with schizophrenia can claim their right to have their end of life needs addressed with compassion and skill.
